# Efficient boundary-guided scanning for high-resolution X-ray ptychography

**DOI:** 10.1107/S1600577523009657

**Published:** 2024-01-01

**Authors:** Dergan Lin, Yi Jiang, Junjing Deng, Fabricio S. Marin, Zichao Wendy Di

**Affiliations:** a Mathematics and Computer Science Division, Argonne National Laboratory, Lemont, IL 60439, USA; b Advanced Photon Source, Argonne National Laboratory, Lemont, IL 60439, USA; Paul Scherrer Institut, Switzerland

**Keywords:** X-ray ptychography, automated data acquisition

## Abstract

In the realm of X-ray ptychography experiments, a considerable amount of ptychography scans are typically performed within a field of view encompassing the target sample. While it is crucial to obtain overlapping scans in small increments over the region of interest for achieving high-resolution sample reconstruction, a significant number of these scans often redundantly measure the empty background within the wide field of view. To address this inefficiency, an innovative algorithm is proposed that introduces automatic guidance for data acquisition. The algorithm first directs the scan point to actively search for the object of interest within the field of view. Subsequently, it intelligently scans along the perimeter of the sample, strategically acquiring measurements exclusively within the boundary of the region of interest. By employing this approach, a reduction in the number of measurements required to obtain high-resolution reconstruction images is demonstrated, as compared with conventional raster scanning methods. Furthermore, the automatic guidance provided by the algorithm offers the added advantage of saving valuable time during the reconstruction process. Through practical implementation on real experiments, these findings showcase the efficacy of the proposed algorithm in enhancing the efficiency and accuracy of X-ray ptychography experiments. This novel approach holds immense potential for advancing sample analysis and imaging techniques in various scientific disciplines.

## Abstract 2

An innovative adaptive scan technique for high-resolution X-ray ptychography is introduced that offers automated guidance for the scan trajectory along an object’s boundary, selectively acquiring measurements relevant to the region of interest.

## 1. Introduction

X-ray transmission ptychography is an imaging technique that can achieve nanoscale spatial resolution, resolving details of the sample that are smaller than the size of the scanning probe (Pfeiffer, 2018 ▸; Suzuki *et al.*, 2014 ▸; Thibault *et al.*, 2008 ▸; Vine *et al.*, 2012 ▸; Wilke *et al.*, 2012 ▸). This high resolution is achieved by combining overlapping measurements of diffraction patterns as the probe scans across the sample within the field of view (FoV) (Bunk *et al.*, 2008 ▸; Takahashi *et al.*, 2011 ▸). In a typical experiment, hundreds or thousands of ptychography scans are collected to achieve large FoV and high-resolution reconstruction (Rodenburg *et al.*, 2007 ▸; Guizar-Sicairos *et al.*, 2014 ▸). While an object’s position is unknown, conventional scanning techniques rely on scanning across a wide FoV that contains the object of interest. This results in spending valuable computational and experimental resources towards the reconstruction of large unimportant regions of an image where the object is absent. An alternative approach involves conducting an initial coarse scan across a wide FoV to gain insight into the object’s dimensions and position. Subsequently, the FoV is refined in a second scan to focus exclusively on the object of interest. In our previous work we have demonstrated an unsupervised learning method that automatically identifies important diffraction patterns measured at scan positions directly across the object within the FoV, and filters out uninteresting background measurements (Lin *et al.*, 2022 ▸). However, this reduction in computational time is limited to the post-processing step, after every measurement is collected within the FoV during the experiment. This leaves much to be desired in terms of optimizing the number of measurements necessary for reconstruction during ptychography experiments to obtain only the important data needed towards reconstruction. In addition to the traditional step scan used in ptychography experiments (Nazaretski *et al.*, 2014 ▸), different scan patterns have been proposed for data collections, such as a spiral pattern to maximize coverage and overlap ratio (Huang *et al.*, 2014 ▸), faster scanning techniques that minimize overheads caused by the settling of piezoelectric scanning systems (Deng *et al.*, 2019 ▸; Pelz *et al.*, 2014 ▸), and using deep reinforcement learning to obtain measurements only at regions that contain repeating atomic structures within the sample (Schloz *et al.*, 2022 ▸). We introduce an adaptive scanning strategy that utilizes a physics-driven method. The strategy aims to guide the scanning probe in locating a specific object within the FoV. By doing so, we can obtain precise measurements solely related to the object itself. This approach ensures that no additional resources are wasted on collecting background data. This also eliminates the need for filtering out and processing ‘uninteresting’ diffraction patterns during the reconstruction. Information regarding feature size, shape and orientation can be extracted from diffraction patterns, and has been used to determine material properties in techniques such as small-angle X-ray scattering (Weinhausen *et al.*, 2012 ▸; Schaff *et al.*, 2015 ▸). However, in our adaptive scan technique, we extract both the scattering strength and direction from the measured diffraction patterns at any given scanned position during the experiment, and guide the scanning probe to the feature change area, consequently guiding the scanning probe to draw a complete perimeter of the region of interest, and finally obtaining measurements within the boundary to obtain a complete dataset that only pertains to the object of interest.

## 2. Methods

Diffraction patterns obtain sample feature information from various perspectives. For example, due to absorption differences, the sum of the pixel intensities of a diffraction pattern measured at an empty background is larger than those measured within a sample. On the other hand, the center of mass (CoM) for each measured diffraction pattern yields the scattering information (in terms of both strength and direction) at the corresponding position of where the diffraction pattern is measured. In a previous work (Lin *et al.*, 2022 ▸), such information has been exploited to automatically identify diffraction patterns that fall within the region of interest (RoI) from all data acquired by a traditional raster-scan approach. As a consequence, only a small fraction of ‘important’ data is used for reconstruction to save overall computational cost. Here, we further extend the applicability of such a CoM feature to actively guide the data acquisition process by directly positioning the scanning beam to the next scan position of interest, thus immediately reducing the experimental cost forefront.

Given a diffraction pattern 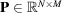 , where *N* × *M* is the size of each diffraction pattern, its two-dimensional CoM along the horizontal direction *x* and vertical direction *y* can be calculated as 

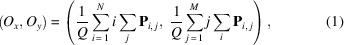

where *Q* is the sum of all pixel values over the entire diffraction pattern, namely, 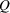 = 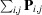 . This two-dimensional CoM indicates the position of the CoM of the diffraction pattern with units of pixels. As an example, a diffraction pattern of size 5 × 5 with uniform intensity throughout all pixels would have a CoM of (3, 3).

In ptychography, calibrating the center of the diffraction pattern is crucial for accurate analysis and reconstruction because it serves as a reference point for aligning and combining the diffraction patterns obtained during the scanning process. Any misalignment or error in the center determination can introduce artifacts and distortions into the extracted information and reconstructed image. In order to calibrate the center of the diffraction pattern, we first obtain *B* diffraction patterns at different known positions of where the object is absent (*i.e.* the background). We can then use (1) to calculate the background CoM 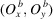 , with (*b* = 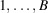 ), for these diffraction patterns. Throughout the paper, we chose *B* = 20. We arrange the calculated background CoM values for these background diffraction patterns into an array, 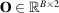 , where row *b* represents the CoM of the *b*th background diffraction pattern. The mean of this background CoM array, 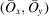 , can then be calculated and used to serve as our ‘calibrated center’ of diffraction patterns. Similarly, the standard deviation of this background CoM array, (σ_*x*_, σ_*y*_), can also be calculated for scaling purposes.

In the final step, we can then represent the new calibrated CoM for each individual diffraction pattern obtained in this experiment as a vector having an origin at the calibrated center, 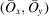 , and becomes unitless after being scaled with the standard deviation, (σ_*x*_, σ_*y*_), as 

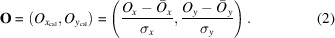

**Figure 1:**
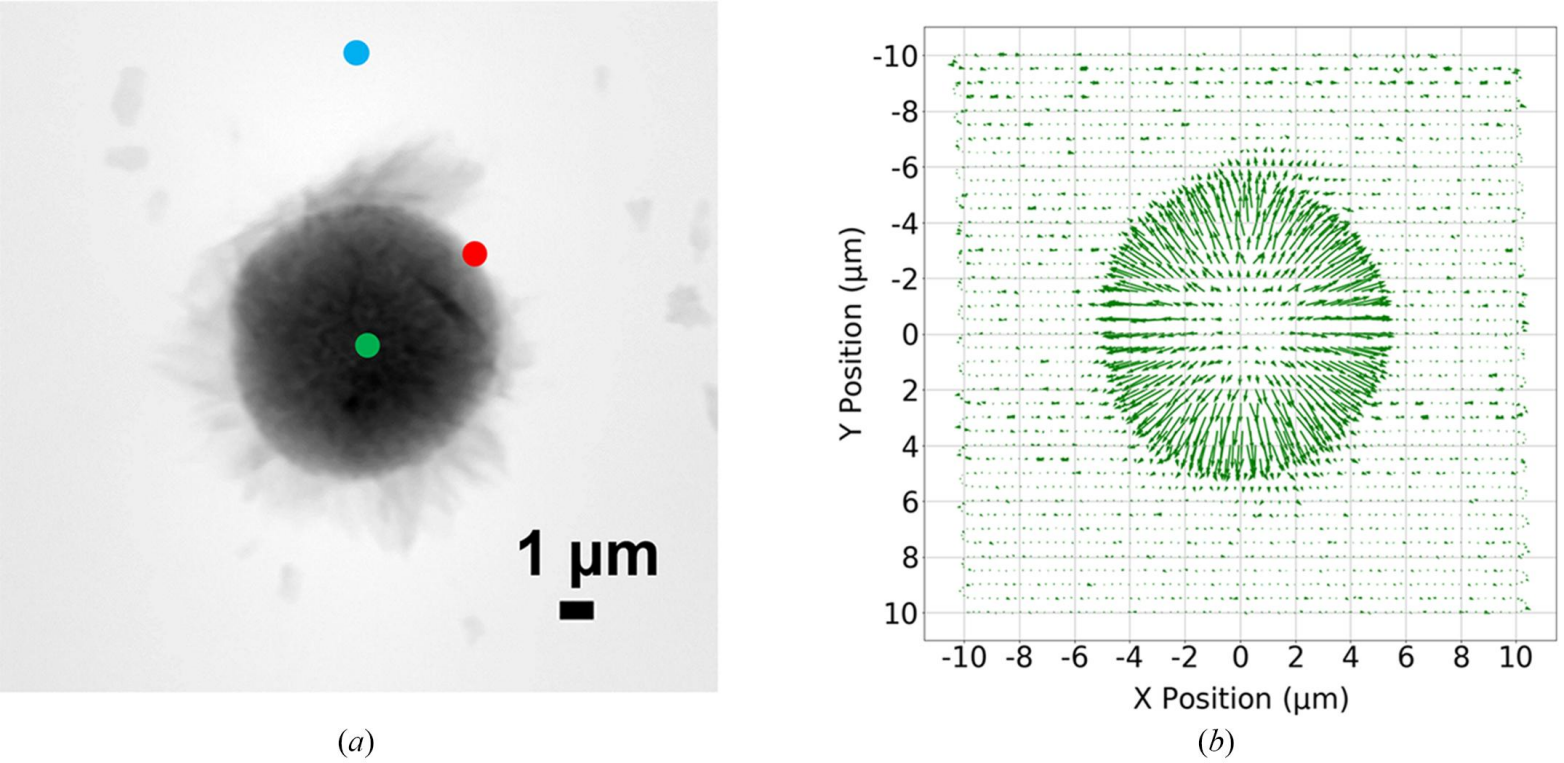
(*a*) Reconstruction of an NCM particle sample. Three diffraction patterns obtained from different scan positions, indicated as colored dots, are presented in Fig. 2 ▸. (*b*) Quiver plot representing the calibrated CoM vector for each diffraction pattern at its scan position, where each arrow provides scattering information (direction and strength). The direction of the arrows points towards the boundary of the object; the length of the arrows along the feature change area is also much more pronounced.

**Figure 2:**
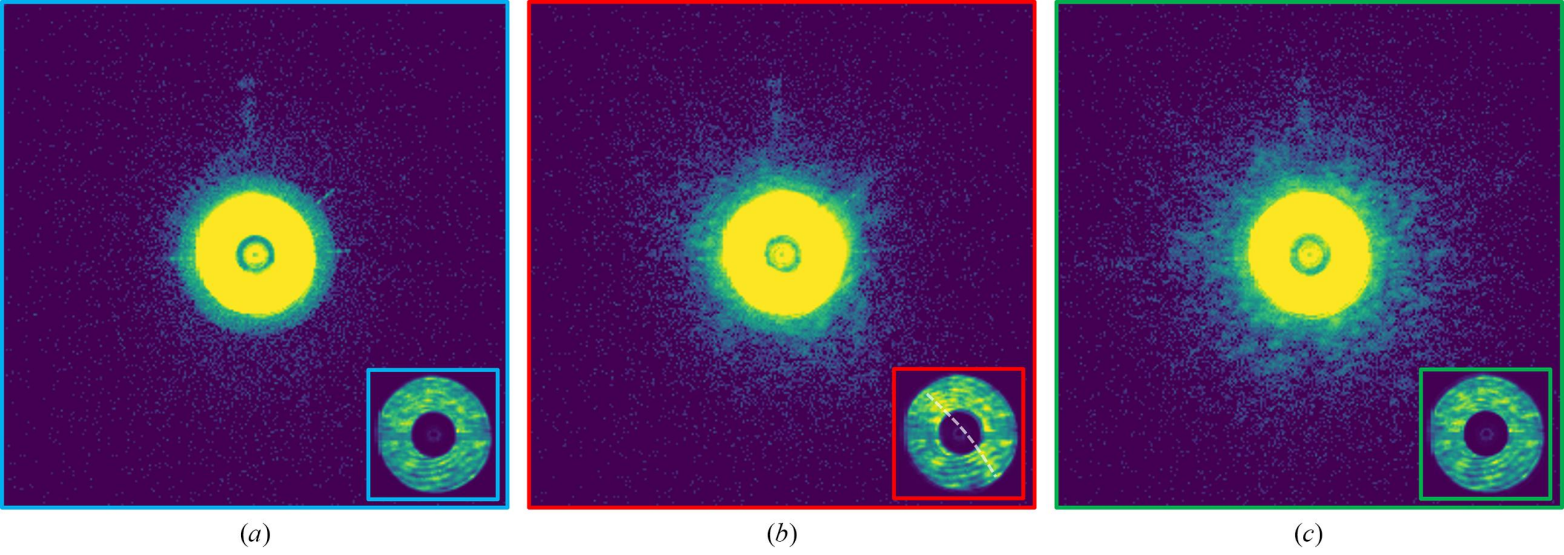
Three examples of diffraction patterns at different scan positions from the sample shown in Fig. 1 ▸(*a*) are presented on a logarithmic scale. (*a*) Empty background (blue). (*b*) On the boundary (red). (*c*) Within the sample (green). The inset in each diffraction pattern shows the central direct beam on a linear scale. The edge feature [marked by a dashed white line shown in (*b*)] is visible when the beam was on the boundary of the particle.

This calibration step helps minimize errors and ensures that the extracted information reflects the true structure of the object under investigation in ptychography. Next we demonstrate how the scattering information (*i.e.* **O**, shown as Fig. 1 ▸) can help identify the RoI. The demonstration is performed on an experimental dataset acquired by the Velociprobe instrument (Deng *et al.*, 2019 ▸) at the Advanced Photon Source. Polycrystalline LiNi_0.6_Co_0.2_Mn_0.2_O_2_ (NCM) cathode particles are imaged at 8.4 keV in the fly scan mode (Deng *et al.*, 2015 ▸), in which the sample is moved continuously as a series of diffraction patterns acquired by an Eiger X 500K detector. A total of 2923 diffraction patterns, covering an area of 21 µm  ×  20 µm, were collected at a continuous frame rate of 200 Hz. Each diffraction pattern was cropped to 128 × 128 and reconstructed by the generalized least-squares maximum-likelihood algorithm (Odstrčil *et al.*, 2018 ▸) implemented in the *PtychoShelves* package (Wakonig *et al.*, 2020 ▸). The reconstructed phase of the sample using the complete dataset is shown in Fig. 1 ▸(*a*), where the corresponding diffraction patterns obtained at the colored positions are shown in Fig. 2 ▸. As shown in Fig. 2 ▸(*b*), edge features that are present only when the scan position is on the boundary of the object provide important scattering information and can be extracted and utilized. This scattering information (CoM), calculated using equation (2), is shown as a quiver plot in Fig. 1 ▸(*b*). Here, each arrow represents the calibrated CoM vector **O** for each diffraction pattern at its measured position; and, as a whole, this provides a preview of the entire map for the scattering information at different positions of the sample.

As illustrated in Fig. 1 ▸(*b*), the scan points within the feature change area (*e.g.* near the particle boundary) exhibit a distinct behavior. In addition to the arrows pointing towards the direction of the RoI boundary (scattering direction), the lengths of the arrows, which correspond to the magnitude of the calibrated CoM vector (*i.e.* ∥**O**∥), are noticeably longer, indicating a higher scattering strength. This observation serves as compelling evidence that the calibrated CoM vector directly conveys information pertaining to the RoI boundary.

The primary objective of our work is to leverage the information directly extracted from the available diffraction patterns, as depicted in Fig. 1 ▸, to facilitate real-time guidance for determining the next scan position. To achieve this, we propose a three-step approach. Firstly, we aim to guide the scanning probe towards a position located at the boundary of the object. Secondly, we intend to direct the probe position along the object’s boundary, thus establishing a complete perimeter. Lastly, our approach involves guiding the probe position to systematically scan the interior of the defined perimeter. These steps collectively enable an efficient and effective scanning strategy based on the information extracted from the existing diffraction patterns.

It is important to note that, for the sake of simplicity, our current work exclusively concentrates on scanning positions defined by discretized grids, governed by fixed horizontal and vertical step sizes denoted as *S*_*x*_ and *S*_*y*_, respectively. However, the algorithm we propose possesses the capability to accommodate various scanning patterns, which will be a focus of our future work. In an initial step, we commence the scanning process by selecting a random straight line in either the horizontal or vertical direction, adhering to the fixed step sizes of *S*_*x*_ or *S*_*y*_, depending on the chosen initial direction.

At each scan position [*X*, *Y*], the diffraction pattern is measured, and the calibrated CoM, 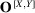 = 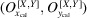 , is updated in real time using the newly acquired data. These updated values are stored for further analysis. To mitigate the effects of noise, a denoising step is incorporated into the process. This involves applying a mean filter after performing a denoise scan, which entails scanning the four neighboring positions. Consequently, the denoised calibrated CoM is represented as 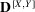 = 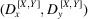 , where 

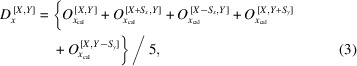




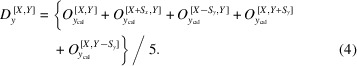

Correspondingly, the denoised scattering strength at position [*X*, *Y*] is defined as 

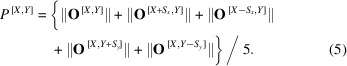

A simple boundary detector can then be implemented by checking whether the denoised scattering strength, *P* ^[*X*, *Y*]^, is greater than a statistically derived threshold, *T*. We use the previously obtained *B* diffraction patterns from empty regions to calculate *T*, as this can provide us with a statistical representation of the scattering strength in empty backgrounds. This can be done by first calculating |**O**| using (2) for each row in **O**, which returns an array of size *B* × 1, and then finding the mean (μ_*b*_) and standard deviation (σ_*b*_) for this array. Finally, we define our threshold *T* = μ_*b*_ + 4σ_*b*_, such that the denoised scattering strength, *P*, at scanning positions along the boundary regions will fall outside the probability limit of belonging to that of an empty region. As we scan along the line chosen in the first step, if no object is detected after the line scan travels beyond reasonable limits, a new line is chosen and repeated until an object is detected with the boundary detector; we then record the diffraction pattern and its scan position as the first position of interest, [*X*_0_, *Y*_0_], and we proceed to the second step.

**Figure 3:**
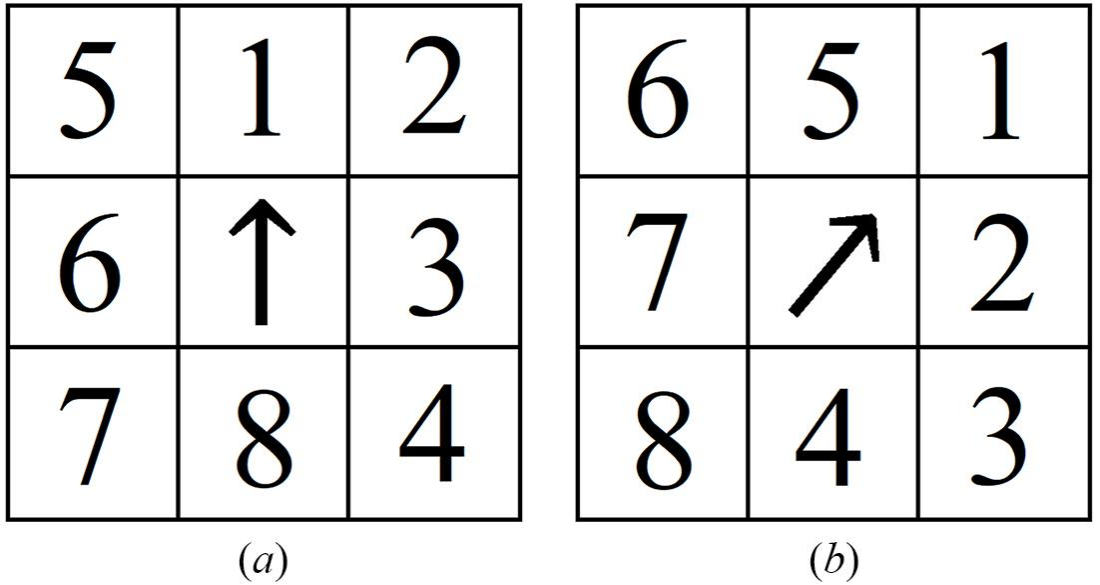
Candidate list for the next scan position based on the direction of the denoised calibrated scattering vector, **D** (shown as the arrow at the current scan position). The candidates are considered as the eight neighboring points of the current position, where the searching starts from the right side of the arrow and moves to the left side of the arrow. (*a*) Example of the priority list positions when the arrow points upwards. (*b*) Example of the priority list positions when the arrow points to the upper right.

The strategy for the second step is to use the direction of the denoised calibrated scattering vector, **D**, to guide the scan point towards the object boundary, and scan along the boundary until a complete loop is drawn as the scan position reaches the position of the first point of interest. To proceed from the current scan position, we iterate the measurements through a candidate list of positions defined as the eight neighboring points of the current position. To be more specific, determined by the scattering arrow of the current position, measurements are first taken on the right half side of the arrow. This is intended to make the scan point move in a clockwise pattern as it progresses along the boundary. Next, measurements are taken on the left half of the arrow. This step accounts for irregular shapes and situations where the boundary does not follow a smooth trajectory. In the rare case of when no adjacent scan positions qualify as a boundary point, an escape routine is executed where the next scan jumps to two steps away to the right of the current position. Fig. 3 ▸ shows an example of the candidate list on a scanning grid, where the numbers indicate the searching order in relation to the arrow direction. Fig. 3 ▸(*a*) shows the the priority list when **D** points upwards, and Fig. 3 ▸(*b*) is an example when the direction of **D** points to the upper right.

As the scanning iterates through the candidate list, whenever the calculated *P* ^new^ measured at a candidate position [*X*_new_, *Y*_new_] passes the boundary detection threshold *T*, it is labeled as a point of interest. The current candidate list then stops iterating, and this new position [*X*_new_, *Y*_new_] becomes the current scan position. The calculated **D** ^new^ then becomes the current **D**, with a new candidate list generated accordingly, and the process is repeated. Note that all measurements made during the candidate list scan and their scan positions are recorded to avoid repeated measurements. This process is repeated until the newly recorded point of interest is within a distance *R* = 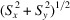 to the first recorded point of interest, [*X*_0_, *Y*_0_], indicating that the scan point has made its way around the perimeter of the object and has completed a closed loop around the RoI. To account for position error in the stepper motor, the stopping criteria distance, *R*, can be adjusted accordingly.

After obtaining the closed perimeter of the object of interest, the final step is to scan the interior of the identified RoI. This step is relatively trivial by scanning through lines between two boundary points back and forth. The pseudo code for the complete algorithm is given below, assuming that the initial scan line will intersect the object.

**Figure 4:**
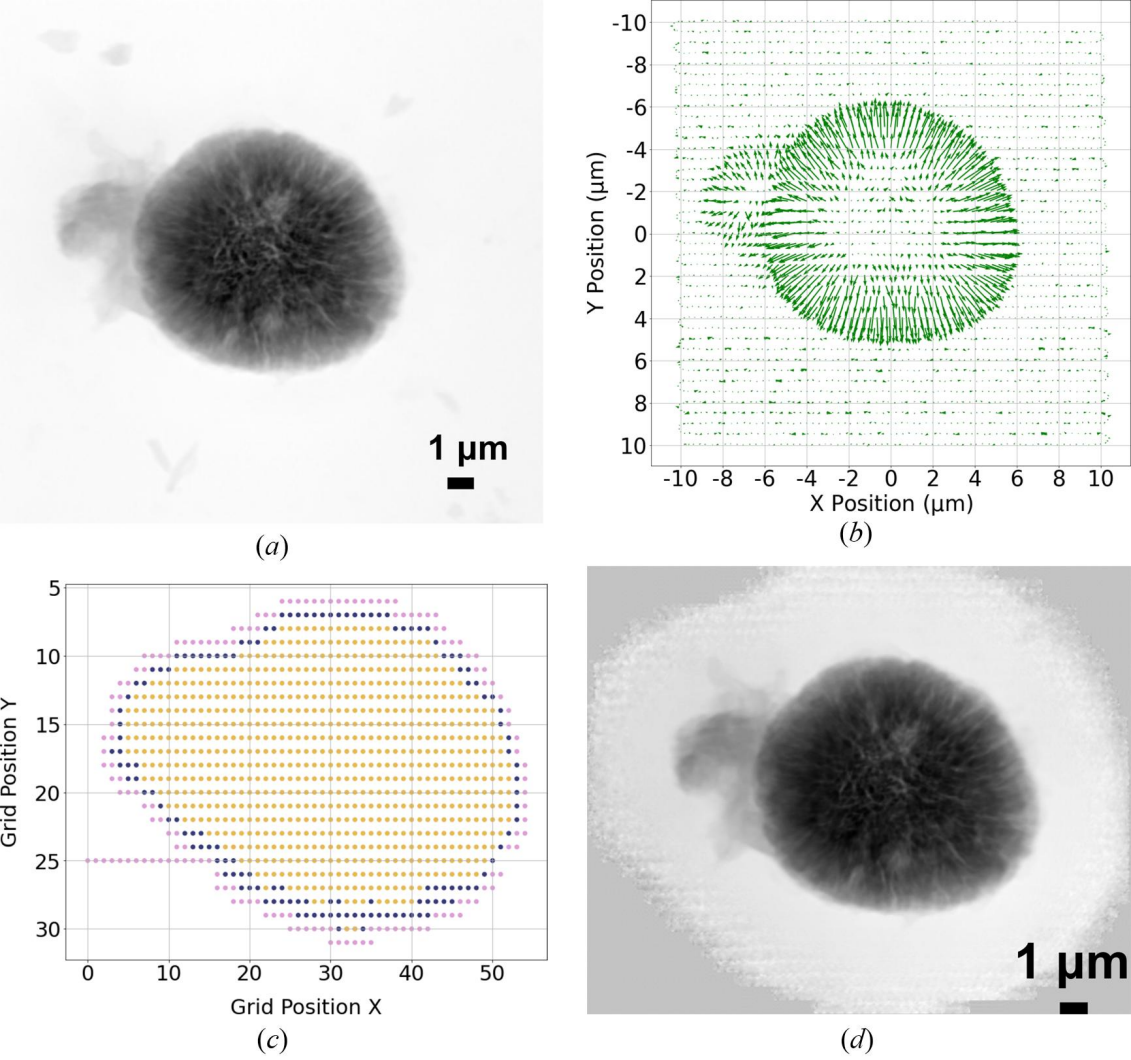
Data obtained from a fly scan experiment for simulation purposes with the following parameters. Horizontal fly scan step size: 300 nm; vertical step size: 500 nm; exposure time at each scan point: 4 ms. (*a*) Object used in the simulation as the reconstruction of a complete dataset from traditional raster scan of the entire FoV. (*b*) Scattering information of the object obtained from each diffraction pattern at their corresponding scan position. (*c*) Simulation results of the adaptive scan algorithm applied on this dataset, where the scan trajectory starts at position [0, 25]. Once the scan point hits the boundary of the object, where the measured *P* ^new^ is greater than *T*, the corresponding scan position is labeled as a point of interest (blue). The adaptive scan algorithm then continues guiding the scan position along the boundary of the object, labeling points of interest as blue, otherwise as pink, until a complete boundary is obtained. The algorithm then continues to scan the points within the obtained boundary, labeled as yellow, to obtain the complete RoI. (*d*) Reconstruction of the object using only the diffraction patterns from the obtained scan positions labeled as the RoI.

**Figure 5:**
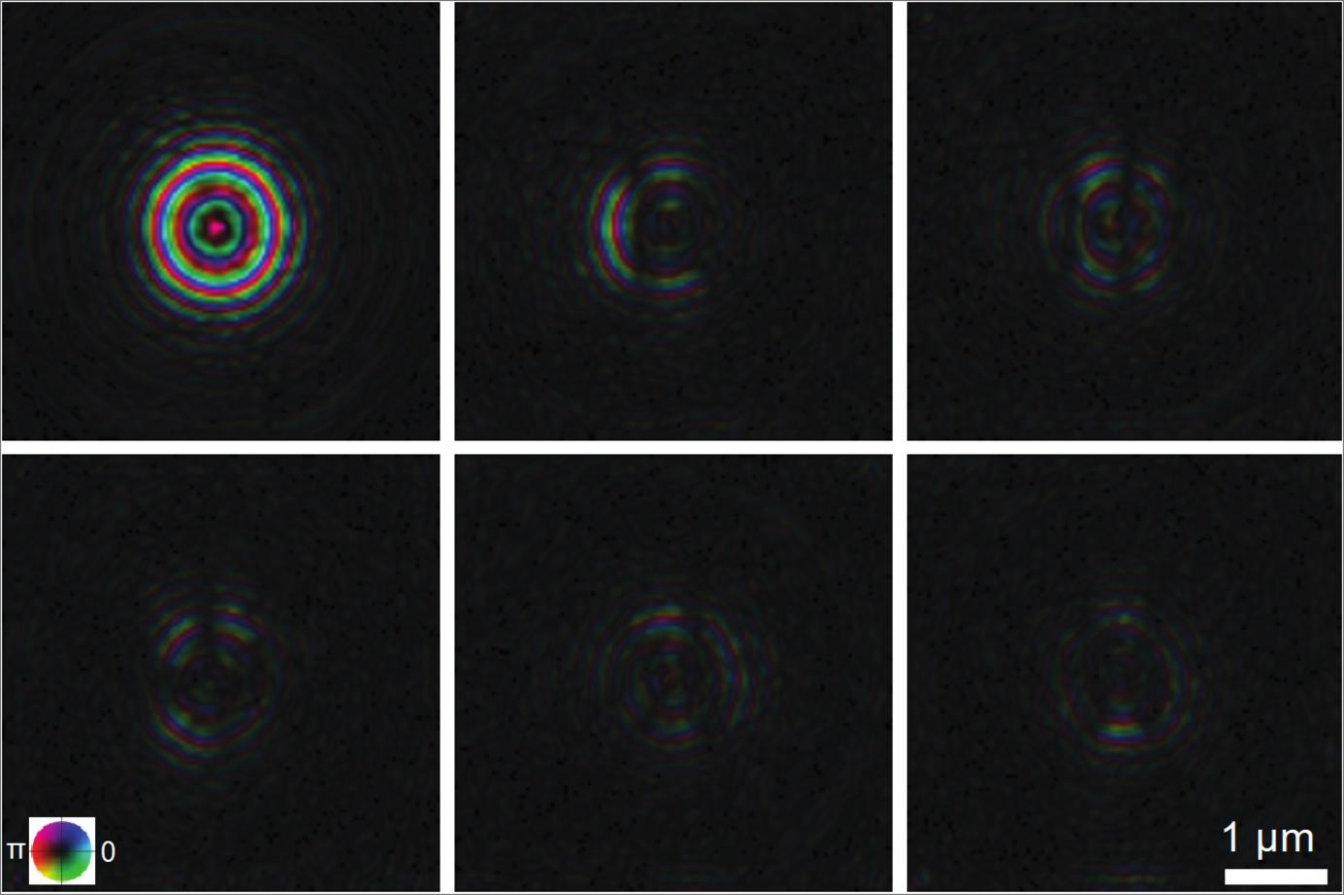
First six primary probe functions of the 15 probe modes used in the reconstruction.

Fig. 4 ▸ demonstrates the simulated result of the proposed algorithm applied on a complete dataset of a different NCM particle sample imaged at 8 keV. The sample-to-detector distance is 2.335 m, and the pixel size of the reconstructed image is 18.9 nm (256 × 256 cropped pixels). The sample was placed about 700 µm downstream of the focus position of the beam, resulting in a full width at half-maximum (FWHM) beam size of 1.2 µm on the sample. Fig. 4 ▸(*a*) shows the object of interest as the reconstruction using all diffraction patterns obtained from a traditional raster scan. Fig. 4 ▸(*b*) shows the scattering information extracted from each scan position in the form of a quiver plot. Fig. 4 ▸(*c*) shows all scanned positions recorded. Starting with the scan position [0, 25], the algorithm guides the scan point in a straight line in search of an object, until it intercepts the object, locating the first scan point where the calculated *P* is greater than *T*, and labels it as a point of interest (blue). The algorithm then guides the scan point along the boundary of the object, where the pink points represent scan positions in the candidate list that were rejected by the boundary detector, and the consequent blue points represent points of interest identified in the candidate list. The boundary searching part stops when it closes the loop and, as a result, identifying points of interest that pertain to the object boundary. A simple algorithm that scans the interior of the obtained boundary is applied, and is represented as yellow points in Fig. 4 ▸(*c*). The yellow points and blue points combined indicate scan positions that fall within the RoI. The reconstruction of the sample using only the diffraction patterns obtained at positions identified within the RoI is shown in Fig. 4 ▸(*d*). Fifteen probe modes were used in the reconstruction: we show the first six primary probe functions in Fig. 5 ▸.

**Figure 6:**
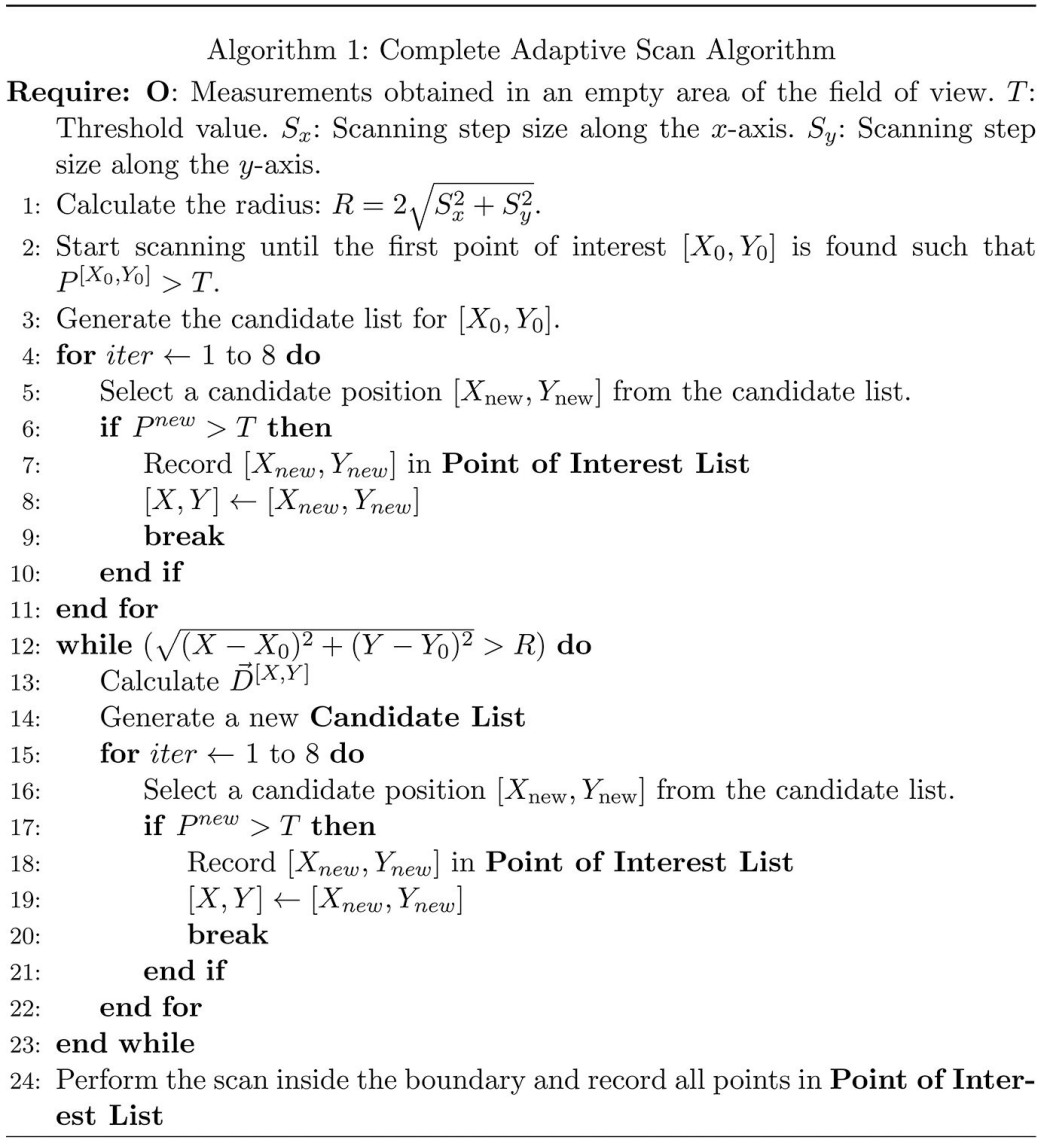
Algorithm 1: complete adaptive scan algorithm.

The complete adaptive scan algorithm is shown in Fig. 6 ▸.

## 3. Experiment

**Figure 7:**
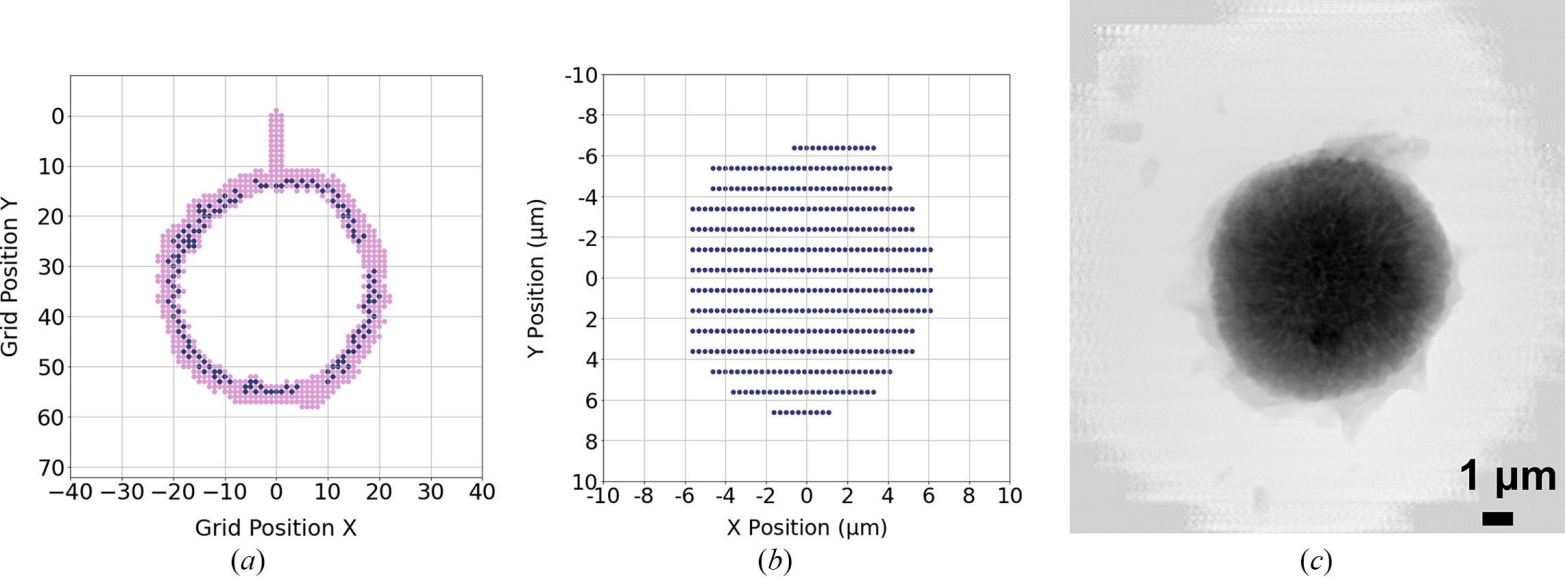
Demonstration of the adaptive scan technique in a real experiment, with 50 ms exposure time at each scan point. (*a*) The scatter plot displays all scan points (677 in total) measured during the experiment. The subset of dark blue points (140 in total) represents the points of interest acquired through the boundary search algorithm, effectively outlining the complete boundary of the object. The horizontal step size, *S*_*x*_, and vertical step size, *S*_*y*_, are both 300 nm. Gaps in the trajectory of the dark blue points correspond to instances when escape jumps are executed. (*b*) Following the boundary determination, a subsequent scan is performed to measure diffraction patterns exclusively within the established object boundary. In this filling scan, the horizontal step is set at 300 nm, and the vertical step size is set at 1000 nm. This scan yields a total of 446 diffraction patterns within the RoI. (*c*) Reconstruction of the object utilizing solely the points obtained within the RoI, resulting in a detailed representation of the object.

We demonstrate the proposed adaptive scan technique in a real experiment on an NCM sample shown in Fig. 1 ▸(*a*). Particles composed of a batch of nanosheets were imaged at 8 keV with the adaptive scan mode (probe size: ∼1 µm in diameter), in which the object is moved continuously as a series of diffraction patterns acquired by an Eiger X 500K detector. Each diffraction pattern was cropped to 256 × 256 and reconstructed by the generalized least-squares maximum-likelihood algorithm (Odstrčil *et al.*, 2018 ▸) implemented in the *PtychoShelves* package. In the experiment, we start by obtaining 20 diffraction patterns at known empty regions, and calculate the statistics needed for the threshold used in the boundary detection. We then initiate the scan along a straight line until intersecting the object. If the scan line does not encounter any particle, it then moves to another scan line by stepping stage *X* or *Y* with a larger step size and continues the scan until encountering a particle. The results are shown in Fig. 7 ▸. In the scatter plot shown in Fig. 7 ▸(*a*), every point corresponds to a scan position that is measured during the experiment. The scan starts from position [0, 0], and travels downwards until the boundary of the object is reached and detected; the corresponding scan position is labeled as a point of interest and marked as blue in the scatter plot. Gaps in the scan trajectory indicate that no adjacent point of interest is found, hence escape jumps are executed. Once the scan position reaches within a neighborhood of the initial point of interest, a complete perimeter is obtained. The algorithm then starts to guide the scan point to obtain diffraction patterns within the determined perimeter, defining the final RoI, shown in Fig. 7 ▸(*b*). Note that the filling pattern used here was a raster scan grid pattern that has been particularly advantageous for maintaining stability during rapid continuous scan; the choice of a filling scan pattern can be tailored to individual requirements, such as concentric and Fermat spiral patterns (Huang *et al.*, 2014 ▸). Fig. 7 ▸(*c*) shows the reconstruction of the object using diffraction patterns that were obtained from scan positions marked in Fig. 7 ▸(*b*). Consequently, a comprehensive scan of a total of 677 points is conducted in order to establish the boundary. Among these, 140 points are designated as points of interest. Additionally, during the process of filling in the obtained boundary, an additional 466 points are scanned, resulting in a combined total of 1143 measurements. In contrast, if we were to consider Fig. 7 ▸(*a*) as an example, employing the conventional raster scan method, 3600 measurements would be taken on a 60 × 60 grid, encompassing every position within the FoV. However, the precision of the FoV on the object would still rely on prior knowledge of its size and shape, necessitating supplementary measurements to acquire such information.

Moreover, as a result of significantly reducing the overall number of measurements in the experiment through the selective measurement of ‘critical’ information, substantial computational resources are conserved during the subsequent reconstruction process, thanks to the reduced volume of experimental data.

## 4. Conclusion

We introduce an innovative adaptive scan technique that offers automated guidance for the scan trajectory along an object’s boundary, selectively acquiring measurements relevant to the RoI. Through comprehensive simulations and real experiments, we have demonstrated the remarkable efficacy of our proposed method. Consequently, the number of required measurements for successful reconstruction is significantly reduced, resulting in substantial savings in computational time during the reconstruction process.

It is important to note that our proposed approach is particularly advantageous for expediting data acquisition on beamlines where a small beam (compared with the scanned sample) is typically employed for numerous scan points. In cases where longer sample-to-detector distances permit the use of a larger beam, the speed improvement demonstrated in our current study may not be as pronounced. However, it is essential to consider that, with the potential to scan larger samples using a larger beam, our approach could still offer valuable benefits, subject to thoughtful planning and optimization.

Our future endeavors involve expanding the current algorithm to encompass the RoI identification of isolated objects within the FoV. To achieve this, we plan to incorporate a coarse scan of the entire FoV, enabling the simultaneous initialization of multiple boundary search processes aimed at identifying these multiple objects. Additional future avenues entail the adaptation of our proposed methodology to various scanning probe dimensions, and the investigation of more resilient scanning patterns for saturating the internal sample region. By implementing these advancements, we can enhance the versatility and applicability of our adaptive scan technique in a broader range of scenarios.
